# Strengthening African health systems through outreach and support and values-driven leadership

**DOI:** 10.4102/phcfm.v13i1.3043

**Published:** 2021-09-06

**Authors:** Louis S. Jenkins, Klaus B. von Pressentin, Zilla North, Gideon van Tonder

**Affiliations:** 1 Department of Family and Emergency Medicine, Faculty of Medicine and Health Sciences, Stellenbosch University, Cape Town, South Africa; 2 Directorate of Primary Health Care, Faculty of Health Science, University of Cape Town, Cape Town, South Africa; 3 Department of Family and Emergency Medicine, Western Cape Department of Health, George Regional Hospital, George, South Africa; 4 Division of Family Medicine, School of Public Health and Family Medicine, Faculty of Health Sciences, University of Cape Town, Cape Town, South Africa; 5 George sub-district, Garden Route District, Western Cape Department of Health, George, South Africa; 6 Riversdale Hospital, Hessequa sub-district, Western Cape Department of Health, Riversdale, South Africa

**Keywords:** Leadership, values, learning culture, family medicine, family physicians, outreach and support, healthcare teams, health systems

## Abstract

Healthcare systems are complex adaptive systems, requiring a change in leadership style, from the traditional model to collaborative, values-driven leadership (VDL). Family physicians are well positioned to facilitate integration and coordination between levels of care, across specialties and within teams, in partnership with local and district management team members. This short report describes a leadership innovation experience in a rural South African district, where a VDL course was introduced in a district health context to build on a strong tradition of relationship-centred outreach and support aimed at creating a learning health system. The authors reflect on the contribution of family physicians to strengthen team-based capacity building, care coordination and a learning culture aimed at quality improvement from the perspectives of the regional and district hospital environments. A values-based leadership style will enable family physicians to strengthen team-relationships and create organisational environments, which support shared learning and quality improvement approaches. Ultimately this approach should lead to improved health systems.

## Introduction

Globally, healthcare organisations have been recognised as complex adaptive systems where traditional leadership models of command and control thinking have shifted towards a collaborative approach.^1^ In complex adaptive systems relationships between parts are more important than the parts themselves.^1^ Health professionals have not been trained in collaborative leadership, which requires one to work across organisational boundaries with a relationship focus to build an environment of trust and mutual respect.^2^ The significance of values-based leadership has subsequently emerged, emphasising care, compassion, trust and respect.^3^ Values-driven leadership (VDL) in healthcare emphasises core values of patient and family centred care, social responsibility and equity.^4^ Values-driven leadership in family medicine (FM) and primary care has been advocated particularly for emphasising the need for collaborative teamwork and good communication skills.^5,6^

Family physicians (FPs) are well positioned to lead in the current healthcare environment, as their generalist training and experience enable them to deal with undifferentiated illness and uncertainty, particularly within the primary health care (PHC) environment.^4,7^ Collaborative leadership between FPs and local managers has been showcased in South Africa (SA), where a supportive environment can enable healthcare workers and the quality of their work to flourish.^8,9^ The coronavirus disease 2019 (COVID-19) pandemic has challenged FPs to extend their leadership roles further in a climate of uncertainty and to navigate complexity without compromising values.^10^ This short report describes leadership innovation in a rural health district in SA, illustrating the contribution of FM to strengthen African health systems.

## Context

The Garden Route district in SA serves 600 000 people through PHC clinics, eight district hospitals, including Riversdale and Mossel Bay hospitals and the George regional referral hospital. It is a rural area with vast distances between facilities, causing many healthcare workers to work in relative isolation. This has health system implications for capacity building and development of healthcare workers, communication and coordination of patient care. As FM was recognised as a specialty in 2007, decentralised postgraduate training strengthened the rural platform through registrars in training and the appointment of one trained FP per subdistrict (a geographic area containing a district hospital and PHC clinics). These FPs lead the local health team in close collaboration with the clinical and medical managers of the subdistrict.

## Outreach and support

The benefits of outreach and support (O&S) from the regional hospital specialists to district hospitals and PHC clinics has been recognised.^11^ For the past 10 years, three regional hospital FPs have been regularly visiting the PHC clinics and district hospital health teams. On average for the period 2015–2019 they conducted 1039 outreach visits, which is 27.2% of the total regional hospital O&S visits for that period (local district health data). During these O&S visits they connected with the local health team of doctors, nurses, allied health professionals and clinical and medical managers. They listened to their workplace dilemmas, whilst building relationships, offering continuing medical education, doing morbidity and mortality reviews, consulting patients in the clinics, wards and emergency centres and operating or giving anaesthetics in the theatres. Whilst communication and good relationships have been highlighted as key elements of effective O&S, most healthcare workers are not comfortable with having difficult conversations or managing dilemmas in the workplace.^11^

## Values-driven leadership

**FIGURE 1:**
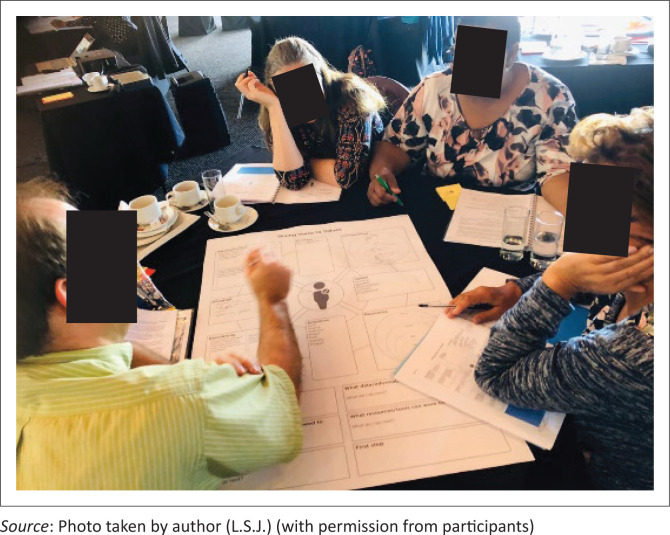
Values-driven leadership workshop participants brainstorming a clinical dilemma: Surgeon, psychiatrist and two nursing managers.

Consequently, an initiative called VDL was introduced in 2019 as a collaboration between the University of Stellenbosch Business School (USB) and the Department of Health. Four weekend workshops were conducted, each with 16–20 participants, for a total of 70 people representing seven hospitals across the region. Participants included doctors, nurses, pharmacists, occupational therapists, physiotherapists, student interns and hospital administrators. Most were in middle or senior management positions or front-line team leaders. Group demographics were diverse and inclusive of gender, race and language. These workshops were facilitated by a consultant at the USB, as well as the medical manager and the head of FM at the regional hospital. Participants brought real health system dilemmas, such as difficulty to refer a patient from a district to the regional hospital and the workshops. Through a process of active listening, appreciative enquiry, various exercises and games, an understanding developed of how VDL drives decisions, policies and culture. Participants were empowered to rescript their thinking and speak out (see Figure 1).

This innovation has enabled integrative thinking, established new relationships and broken down silos and hierarchies in the health system, by making explicit the shared values of care, compassion, accountability, integrity, respect and responsiveness. During 2020, with the disruption of the COVID-19 pandemic, face-to-face workshops were replaced with monthly discussions around ethical issues in the workplace via online Microsoft (MS) Teams conversations between district and regional hospitals. Research, measuring the impact of the VDL initiative is ongoing, with initial results 1 year later showing the value of understanding the ‘me-we-all of us’ perspective.^7,12^ In other words, participants began to understand how individual actions are determined by underlying values and how these values drive professional practice within their team and ultimately influence the health system. This happened through processes of active, respectful listening and relationship building. Finally, a sense of how individual leadership and teamwork fitted into the health system emerged. Box 1 provides an example of a weekend workshop participant’s reflection.

BOX 1 Family physician reflection on a weekend values-driven leadership workshop.

During the values-driven leadership (VDL) workshop, we were provided with a template to ‘give voice to values’ (finding values-based solutions to real challenges experienced at work). The dilemma I experienced revolved around the challenges encountered whilst striving to create a learning environment for the family medicine residents (postgraduate students or registrars) in the district hospital’s clinical environment. There were varying degrees of buy-in and support from the clinical team, oscillating between the need to get the clinical work done and the need to allow these residents access to clinical training opportunities. The underpinning values at play here were *responsibility* (whose responsibility is it to ensure the success of the residency programme) and *fairness* (how do I balance this with the need to ensure that other members of the team also experience capacity building opportunities). The workshop facilitators and fellow attendees, as well as the VDL template, allowed me to explore the dilemma and unlock the situation, by rescripting my response to the dilemma (e.g. by acknowledging that I as the family physician am not solely responsible for creating a structured learning environment, and that I should allow the clinical team to share this responsibility). This ‘rescripting’ approach was useful to develop a fresh approach to the dilemma. I resolved to take a more appreciative approach, to engage constructively with the clinicians and managers and to focus on the relationships within the team. By being advised to ‘hold it lightly’, it enabled me to allow for ‘things to develop more organically’ in a collaborative manner built on conversations around shared learning needs and thoughts on how best to strengthen the service and workspace learning environment.

## Discussion

Through the VDL programme FM is playing an active role to help create a culture of learning and reflection in the health system. The weekend workshops and monthly MS Team meetings have allowed relationships to strengthen, communications to improve and appreciation of the value of various team members to grow. Family physicians and health managers who live according to the principles of FM have enabled traditional hierarchical and mechanistic organisational culture to slowly transform.^7,13^ These processes made people more aware of alignment between their personal values and behaviour and organisational values and behaviour (processes, rules and procedures). Dilemmas were resolved by being clearer about alignment or non-alignment with values that were more clearly articulated, and therefore were easier to solve. The end result of leadership capacity building within various health teams through the VDL programme and relationship building between people in different subdistricts through O&S is improved coordination of care for patients.^3^ It follows that apart from the role of the FPs as an expert clinician, being a capacity builder with a district health system perspective is a vital role to ensure best care for patients in the communities they serve.

The contribution of FM to the health system may be found in the following themes: as capacity builders, the FPs at George and Mossel Bay hospitals collaborated closely with the local medical and clinical managers to facilitate the capacity development of healthcare workers in the district health system. The years of relationship building during O&S, and recently through the VDL workshops across subdistricts, have enabled communication to become respectful, caring and responsive to the real needs of patients and providers. The FPs are in a unique position to consider the whole health system, as the discipline cares transversally for patients and staff across the platform from PHC to district hospital and to every clinical discipline in the regional hospital.^8^ Consequently, FPs coordinate the O&S programmes, and more recently the outreach and in-reach programmes, bringing medical officers, especially community service medical officers new to the district, into the regional hospital for a week, whilst supporting the district hospital with an outreaching medical officer from the regional hospital to build relationships, expand contexts and develop clinical skills.

The FP positioned at the regional hospital, where specialist care is predominantly structured in siloed departments, delivers transversal roles, including those being responsible for clinical governance and capacity building, coordinating services that are relevant to all departments and therefore owned by none. At George Hospital, the FM Department has been instrumental in the development of the antibiotic stewardship programme,^14^ the palliative care service,^15^ the emergency centre service, the institutional training programme, the women’s health and Thuthuzela (Sexual Assault) service, the response to tuberculosis, occupational health, wellness and a smoking cessation clinic for staff. The department has also been the driving force behind breaking down hierarchies between professions, inviting the pharmacists and the infection and prevention control nurse to the antibiotic stewardship rounds, introducing whole person learning, as two examples.

It is within these roles of the FP where good communication skills and collaborative teamwork within the complexity of the health system create a different experience of patient care, which reflects in patient satisfaction and community trust in the health service. Because FPs function in more than one clinical domain, with a number of roles beyond that of being a clinician, their integrative role can often be mistaken for assimilation into the main clinical disciplines or simply role confusion. It remains a challenge to maintain identity as FPs, whilst leading collaboratively through integrated patient care within a complex health system. Here the roles of the FP in the district hospital, where there are no clinical departments, are arguably simpler, but not less challenging, as has been described previously.^16^

## Conclusion

A targeted values-based leadership training intervention helped to strengthen the relationships between healthcare workers and managers at different care levels within a shared geographic space, formed over years of outreach and support by specialists, including FPs. A values-based leadership style will enable FPs to strengthen team-relationships and create organisational environments, which support shared learning and quality improvement approaches. Ultimately this approach should lead to improved health systems.
